# Routine Biomarkers in Paediatric Appendicitis Stratification: Which Add Diagnostic Value? A Retrospective Cohort Study

**DOI:** 10.3390/children13040447

**Published:** 2026-03-25

**Authors:** Ciprian-Ioan Borca, Alexandru Alexandru, Madalin-Marius Margan, Cristiana-Smaranda Ivan, Alexandru Cristian Cindrea, Corneluta Fira-Mladinescu, Marius Negru, Delia Hutanu, Silviu-Valentin Vlad, Brigitha Vlaicu, Vlad-Laurentiu David

**Affiliations:** 1 Doctoral School, Victor Babes University of Medicine and Pharmacy, E. Murgu Square, No. 2, 300041 Timisoara, Romania; 2 Department of Paediatric Surgery and Orthopaedics, “Louis Turcanu” Emergency Children’s Hospital, 300011 Timisoara, Romania; david.vlad@umft.ro; 3 Discipline of Public Health, Department of Functional Sciences, Victor Babes University of Medicine and Pharmacy, 300041 Timisoara, Romania; 4 Centre for Translational Research and Systems Medicine, Faculty of Medicine, Victor Babes University of Medicine and Pharmacy, 300041 Timisoara, Romania; 5 Emergency Department, Emergency Clinical Municipal Hospital, 300254 Timisoara, Romania; 6 Department of Microbiology, Discipline of Hygiene, Victor Babes University of Medicine and Pharmacy, 300041 Timisoara, Romania; 7 Centre for Studies in Preventive Medicine, Victor Babes University of Medicine and Pharmacy, 300041 Timisoara, Romania; 8 Department of Biology, Chemistry-Biology-Geography Faculty, West University of Timisoara, Pestalozzi Street, No 16 A, 300115 Timisoara, Romania; 9 Department of Surgery, Faculty of Medicine and Pharmacy, University of Oradea, 410073 Oradea, Romania; 10 Department of Paediatric Surgery and Orthopaedics, Victor Babes University of Medicine and Pharmacy, 300041 Timisoara, Romania

**Keywords:** paediatric, acute appendicitis, complicated appendicitis, biomarkers, C-reactive protein, fibrinogen, systemic immune-inflammation index (SII), neutrophil-to-lymphocyte ratio (NLR)

## Abstract

Background: Preoperative differentiation between uncomplicated and complicated paediatric appendicitis remains challenging. This study aimed to evaluate the diagnostic performance of routine admission biomarkers and blood cell count-derived inflammatory indices for severity stratification and to determine whether fibrinogen provides additional predictive value beyond commonly used markers. Methods: We conducted a retrospective single-centre study (2018–2025) using electronically recorded clinical data. Patients with suspected appendicitis were identified through appendicitis-related ICD-10 codes and diagnostically validated. The final analytical cohort required complete admission laboratory data, including C-reactive protein (CRP), fibrinogen, and complete blood count parameters. Derived inflammatory indices included the neutrophil-to-lymphocyte ratio (NLR) and the systemic immune-inflammation index (SII). Diagnostic discrimination and multivariable prediction models were evaluated to assess the ability of these markers to distinguish complicated from uncomplicated appendicitis. Results: Of 1518 screened records, 1132 patients met inclusion criteria (620 complicated; 512 uncomplicated). Complicated appendicitis was associated with higher inflammatory markers and longer hospital stay (all *p* < 0.001). CRP demonstrated the strongest univariable discrimination (area under the curve [AUC] 0.785), while fibrinogen showed lower performance (AUC 0.744). A combined model including CRP, NLR, and SII achieved good discrimination (AUC 0.812), with minimal improvement after adding fibrinogen (AUC 0.813). In multivariable analysis, log-transformed CRP and SII remained independently associated with complicated appendicitis (both *p* < 0.001). A rule-out probability threshold of 0.303 achieved 90% sensitivity (negative predictive value 0.803), whereas a CRP cut-off ≥92.24 mg/L showed high specificity (0.943) and positive predictive value (0.900). Conclusions: Routine admission biomarkers and inflammatory indices derived from complete blood counts can support severity stratification in paediatric appendicitis. CRP and SII provide meaningful predictive information, whereas fibrinogen contributes little additional discriminatory value beyond CRP-based models. These findings suggest that a small set of routinely available laboratory markers may assist early risk stratification, although external validation is required before clinical implementation.

## 1. Introduction

Acute appendicitis (AA) represents the most common surgical emergency in paediatric patients, characterised by the abrupt inflammation of the vermiform appendix, often requiring urgent intervention to prevent severe complications [1,2]. In children, AA is categorized into uncomplicated (UA) and complicated (CA) forms, with UA involving localized inflammation without perforation or gangrene, while CA progresses to necrosis, perforation, abscess formation, or peritonitis, potentially leading to sepsis and increased morbidity [3,4].

Although the distinction between UA and CA is widely accepted, formal definitions vary slightly across guidelines and major trials. The European Association for Endoscopic Surgery and the World Society of Emergency Surgery define complicated appendicitis primarily by the presence of gangrene or perforation, often accompanied by abscess, phlegmon, or purulent peritonitis [5,6]. Similarly, contemporary clinical studies describe uncomplicated appendicitis as inflammation without radiologic or clinical evidence of perforation or abscess, whereas complicated disease includes rupture, diffuse peritonitis, severe phlegmon, or systemic septic manifestations [7,8]. Despite minor variations, the shared defining feature of complicated appendicitis remains transmural necrosis and/or perforation with local or generalized infectious complications.

The distinction is important, as UA in paediatrics is managed conservatively with antibiotics, reducing surgical risks, hospital stays, and costs, whereas CA typically necessitates prompt appendectomy, either open or laparoscopic, given the rapid progression to perforation—often within 24–48 h of symptom onset [9,10]. Inflammatory mass with or without non-drainable fluid collection <3 cm, also known as peri-appendiceal mass, is worth mentioning as being considered a particular type of complicated appendicitis, though some authors describe it as a mass without further detailing [11,12]. Accurate preoperative differentiation remains challenging due to variable clinical presentations in children, who exhibit nonspecific symptoms like abdominal pain, vomiting, or fever, complicating timely diagnosis [2,13,14].

To address this diagnostic challenge, recent research has emphasized refining the biochemical profile in paediatric acute appendicitis [15,16]. Beyond traditional markers such as C-reactive protein (CRP) and leucocytosis, several parameters show promise for severity stratification [17,18]. These include largely available parameters such as fibrinogen and international normalized ratio (INR), alongside composite ratios from routine blood counts—neutrophil-to-lymphocyte ratio (NLR), platelet-to-lymphocyte ratio (PLR), monocyte-to-lymphocyte ratio (MLR), systemic immune-inflammation index (SII), and pan-immune-inflammation value (PIV)—which offer a more detailed view of systemic inflammation [18,19,20]. Specific patterns provide clinical insights: an elevated RDW/Hb ratio may signal early systemic stress [21], while a decreased haemoglobin-to-platelet ratio (HPR) has been linked to antibiotic treatment failure [22]. Nevertheless, optimal diagnostic thresholds for these indices remain unclear in children, and the independent value—or potential redundancy—of traditional markers like fibrinogen requires further clarification.

Imaging modalities play an important role in evaluation, with ultrasound (US) recommended as the first-line tool by the World Society of Emergency Surgery (WSES) for its accessibility, lack of radiation, and ability to detect signs of perforation or abscess in children [23]. Computed tomography (CT) is reserved for equivocal US findings due to radiation concerns, while magnetic resonance imaging (MRI) offers high sensitivity for complications but is limited by availability and potential contrast-related risks like allergic reactions or nephrotoxicity [23,24]. Clinical scoring systems, such as the Alvarado score, Paediatric Appendicitis Score (PAS), and Appendicitis Inflammatory Response (AIR) score, incorporate symptoms, signs, and basic labs but often fall short in reliably distinguishing UA from CA in paediatrics [25,26]. Advanced scales integrating imaging and biomarkers, like the Acute Appendicitis Severity Index (AASI) or Atema score, aim to address these gaps, yet no universal consensus exists, partly due to population-specific variability [27,28]. The postoperative histopathology report thus serves as the gold standard for confirmation [29].

In parallel with these traditional diagnostic strategies, recent years have seen increasing interest in artificial intelligence (AI) and machine learning–based approaches for the diagnosis and severity stratification of acute appendicitis. A recent comprehensive review highlights the growing role of AI-driven algorithms in predicting appendicitis severity and guiding clinical decision-making [30]. However, many AI-based models rely on complex architectures, require large, annotated datasets, and may be difficult to implement in routine clinical workflows. In contrast, prediction strategies based on routinely available laboratory biomarkers offer transparency, simplicity, and immediate applicability at the point of care.

The primary objective of this study was to evaluate the diagnostic utility of routine inflammatory markers (e.g., CRP, NLR, PLR, MLR, and related indices) in distinguishing UA from CA, including perforated cases, in paediatric patients, to support improved preoperative severity stratification and management decisions. As a secondary objective, we assessed whether fibrinogen provides independent predictive value beyond these established routine markers or is redundant in the assessment of paediatric appendicitis severity.

## 2. Materials and Methods

### 2.1. Study Design and Patient Selection

This retrospective observational study was conducted at the “Louis Țurcanu” Emergency Children’s Hospital in Timișoara, Romania, using electronic health records (EHRs) from 1 January 2018 to 31 December 2025. The primary objective was to evaluate the diagnostic utility of routine admission laboratory parameters in differentiating between complicated and uncomplicated appendicitis, as well as predicting perforation status. Initial screening identified paediatric patients using ICD-10 codes associated with appendiceal inflammation: K35.9(acute appendicitis), K36 (other appendicitis), K37 (unspecified appendicitis), and K38.8 (other specified diseases of the appendix) [31].

To maintain a standardized analytical cohort, a deduplication protocol was applied to patients with multiple admissions. Records were reconciled using an attributed unique patient identifier (PI); in cases of record duplication, a single entry was retained, while in cases of differing admission of the same patient, the admission corresponding to the pre-appendicectomy moment was preserved to capture the period of peak inflammatory response [32]. Following initial identification, a secondary manual review of surgical and pathological reports was performed to exclude cases where appendicitis was not post-operatively or clinically confirmed (e.g., negative appendectomies or alternative diagnoses).

### 2.2. Inclusion and Exclusion Criteria

Eligibility was restricted to patients with a confirmed diagnosis of appendicitis. Inclusion required the availability of a complete baseline laboratory profile obtained at the time of admission. Specifically, patients were required to have documented values for: white blood cell count (WBC), haemoglobin (HGB), absolute neutrophil count, absolute lymphocyte count, and fibrinogen.

A complete-case approach was utilized, and records with any missing laboratory parameters were excluded from the final analysis. While this ensures a robust dataset for multivariate modelling, it assumes data are missing completely at random (MCAR) [33]. Patients with pre-existing haematological disorders or chronic inflammatory conditions that might confound baseline laboratory markers were also excluded.

### 2.3. Outcome Definitions and Variables

The primary outcomes were the classification of appendicitis as “complicated” or “uncomplicated” and the identification of “perforated” status. Patients are classified as having uncomplicated or complicated appendicitis based on pre-operative, intra-operative, and/or histopathological findings. Uncomplicated appendicitis is defined as an inflamed appendix without evidence of gangrene, perforation, intraperitoneal purulent fluid, contained peri-appendiceal phlegmon, or intra-abdominal abscess. Complicated appendicitis includes all cases featuring a gangrenous, inflamed appendix (with or without perforation), intra-abdominal abscess, peri-appendicular contained phlegmon, or purulent free fluid, in accordance with the European Association for Endoscopic Surgery (EAES) consensus development conference 2015 [5]. Histopathological reports were reviewed for all included cases to confirm the diagnosis of appendicitis; patients in whom appendicitis was not confirmed were excluded from the final cohort. In cases where discrepancies existed between operative and pathological descriptions, the presence of any features indicating advanced disease—such as gangrene, perforation, intra-abdominal abscess, peri-appendiceal phlegmon, or purulent intraperitoneal fluid—resulted in classification as complicated appendicitis.

The independent variables included demographic data (age and sex), admission laboratory markers, and length of hospital stay. All laboratory parameters analysed were obtained as part of routine admission blood tests performed during the standard diagnostic work-up for suspected appendicitis; no laboratory investigations were ordered specifically for research purposes. Derived inflammatory indices, such as the Neutrophil-to-Lymphocyte Ratio (NLR), Platelet-to-Lymphocyte Ratio (PLR), Lymphocyte-to-Monocyte Ratio (LMR), and Systemic Immune-Inflammation Index (SII), were calculated from complete blood count parameters to evaluate systemic inflammatory response.

### 2.4. Ethical Considerations

The study protocol was reviewed and approved by the Ethics Committee of the “Louis Țurcanu” Emergency Children’s Hospital (Approval No. 2860). All procedures adhered to the principles of the Declaration of Helsinki [34]. In accordance with the General Data Protection Regulation (GDPR), all patient data were de-identified prior to statistical processing [35]. A waiver of informed consent is acceptable and granted due to the retrospective, non-interventional nature of the study.

### 2.5. Statistical Analysis

Data were first anonymized and prepared for analysis using Python (v 3.13.2) with the pandas (v. 2.2.3) package for data manipulation, cleaning, and derivation of composite inflammatory indices (NLR, PLR, LMR, SII). All subsequent statistical analyses were conducted using IBM SPSS Statistics (version 26; IBM Corp., Armonk, NY, USA).

Continuous variables are presented as medians with interquartile ranges (IQRs) and, where informative, as means with standard deviations (SDs). Normality of continuous variables (age, length of hospital stay, laboratory parameters, and derived inflammatory indices) was assessed using the Shapiro–Wilk test. All evaluated variables demonstrated significant deviation from normality (*p* < 0.001). Categorical variables are summarized as absolute frequencies and percentages.

Univariable comparisons between complicated and uncomplicated appendicitis groups were performed as follows: for continuous variables with non-normal distribution (age, length of hospital stay, haematological parameters, CRP, fibrinogen, and composite indices), the Mann–Whitney U test was applied. Categorical variables (sex and appendicectomy status) were compared using the chi-square test.

Multivariable logistic regression was used to identify independent predictors of complicated appendicitis, with adjustment for confounders. Two models were fitted: a primary model containing log-transformed CRP, log-transformed SII, NLR, age (years), and binary sex; and a secondary model substituting log-transformed fibrinogen for CRP. Continuous predictors were log-transformed where skewed to improve linearity and standardized (z-scores) for effect-size comparability and numerical stability. Class weighting was applied to account for moderate outcome imbalance. Model fit and discrimination were evaluated using AUC, AIC, and Nagelkerke pseudo-R^2^. Nested comparisons (likelihood-ratio tests and ΔAIC) assessed the added value of fibrinogen, especially for perforation prediction. Cross-validation provided estimates of out-of-sample performance and incremental discriminative gain (ΔCV-AUC). Specifically, stratified 10-fold cross-validation was applied, with folds constructed to preserve the proportion of complicated-to-uncomplicated cases within each partition. In each iteration, model coefficients and standardisation parameters were re-estimated on nine folds, and predictions were generated on the held-out tenth fold. Cross-validated AUC was computed from pooled out-of-sample predictions aggregated across all ten held-out folds, and ΔCV-AUC was calculated as the difference in CV-AUC between nested models.

Diagnostic thresholds were derived from the primary multivariable model probabilities and standalone CRP values. The Youden index (sensitivity + specificity − 1) identified the optimal balanced probability threshold. A lower threshold was selected to target ≥90% sensitivity for rule-out purposes. A high-specificity CRP threshold was chosen to achieve ≥90% PPV for rule-in decisions. Sensitivity, specificity, positive predictive value, negative predictive value, and accuracy were calculated at each threshold using standard confusion matrix methods on the complete-case analytical cohort. All tests were two-sided, with statistical significance defined as *p* < 0.05.

During the preparation of this manuscript, the authors employed ChatGPT (GPT-5.2.; OpenAI, San Francisco, CA, USA) solely to assist with language editing and clarity of expression, to ensure better readability in the context of the authors being non-native English speakers. The study design, data analyses, interpretation of findings, and all scientific conclusions were developed independently by the authors, who assume full responsibility for the content.

## 3. Results

Initial electronic health record (EHR) screening identified 1518 records associated with appendicitis-related ICD-10 codes. Following the deduplication protocol, 105 duplicate admissions (6.9% of the original total) were removed, leaving 1413 unique patient records.

Subsequent diagnostic validation through clinical and pathological review led to the exclusion of 37 patients (2.6% of the deduplicated group) whose presentation did not align with a confirmed diagnosis of appendicitis. Of these excluded cases, 30 patients (81.1% of exclusions) were ultimately diagnosed with mesenteric adenopathy, a common clinical mimic of paediatric appendicitis. The remaining 7 cases involved alternative intra-abdominal pathologies initially misidentified during the preliminary coding phase.

**Figure 1:**
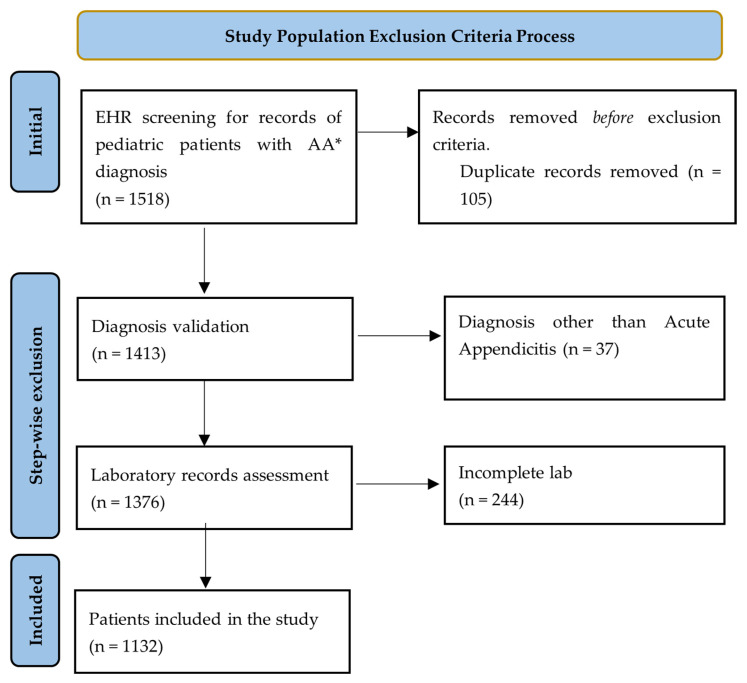
Study population flowchart. * AA—Acute Appendicitis.

Of the 1376 patients with confirmed appendicitis, 244 (17.73%) were excluded due to incomplete laboratory datasets, specifically missing admission values for CRP, INR, WBC, HGB, neutrophils, lymphocytes, or the highest contributor, fibrinogen. The application of this complete-case approach resulted in a final analytical cohort of 1132 patients (82.26% of the confirmed appendicitis group). The selection process with the exclusion steps can be visualised in Figure 1.

### 3.1. Study Population—General Characteristics

In the analysis of the final analytical cohort (*n* = 1132), significant differences were observed in age, sex distribution, and clinical course between patients with complicated (*n* = 620, 54.8%) and uncomplicated (*n* = 512, 45.2%) appendicitis.

The median age of the entire cohort was 11.12 years (IQR 8.58–14.42). Patients in the complicated group presented with a lower median age (10.67 years; IQR 8.06–14.42) compared to those in the uncomplicated group (11.50 years; IQR 9.08–14.44). This difference in age distribution was statistically significant (MWU, *p* = 0.01).

The cohort exhibited a male predominance, with 719 males (63.5%) and 413 females (36.5%). Although a higher proportion of males was observed in the complicated group (66.1%) compared to the uncomplicated group (60.4%), the difference in sex distribution did not reach statistical significance (ChiSq, *p* = 0.05).

The median length of stay for the entire cohort was 6.0 days (IQR 4.0–8.0). Patients with complicated appendicitis experienced significantly longer hospitalizations (Median 8.0 days; IQR 6.0–9.0) compared to those with uncomplicated disease (Median 4.0 days; IQR 3.0–6.0). The difference was highly statistically significant (MWU, *p* < 0.001). Appendicectomy rates also significantly differed between the two subgroups (ChiSq, *p* = 0.001).

In terms of representativeness of the final cohort based on characteristics up until this point, age, sex, and length of stay (los) did not present a significant statistical difference between the final cohort and the excluded cohort (age MWU, *p* = 0.134; sex ChiSq, *p* = 0.413; los MWU, *p* = 0.710). Disease severity reached significance (ChiSq, *p* = 0.006). Table 1 contains summarised population characteristics.

### 3.2. Laboratory Parameters and Inflammatory Biomarkers

Comparative analysis of admission laboratory data demonstrated distinct profiles between complicated and uncomplicated appendicitis, reflecting varying degrees of systemic inflammatory response and physiological stress.

Patients with complicated appendicitis exhibited significantly higher median white blood cell (WBC) counts (17.71 × 10^3^/μL) and absolute neutrophil counts (14.50 × 10^3^/μL) compared to those with uncomplicated appendicitis (14.05 × 10^3^/μL and 10.89 × 10^3^/μL, respectively; *p* < 0.001 for both). Conversely, absolute lymphocyte counts were significantly lower in the complicated group (1.39 × 10^3^/μL) than in the uncomplicated group (1.92 × 10^3^/μL; *p* < 0.001) (Table 2).

Biochemical markers of inflammation, CRP and fibrinogen, exhibited the most pronounced differences between the groups. Median CRP levels were approximately fivefold higher in patients with complicated appendicitis (75.39 mg/L) compared to those with uncomplicated appendicitis (15.04 mg/L; *p* < 0.001). Fibrinogen concentrations were similarly elevated in the complicated group (433.00 mg/dL) relative to the uncomplicated group (345.00 mg/dL; *p* < 0.001) (Table 3).

### 3.3. Composite Inflammatory Indices and Predictive Modelling

Beyond individual haematological parameters, composite inflammatory indices were calculated to assess the balance between innate and adaptive immune responses. All evaluated indices—NLR, PLR, LMR, and SII—differed significantly between groups (*p* < 0.001 for all). Patients with complicated appendicitis exhibited markedly higher median NLR (10.38 vs. 5.38) and SII values (3094.92 vs. 1560.39), consistent with a more intense systemic inflammatory burden (Table 4).

**Figure 2:**
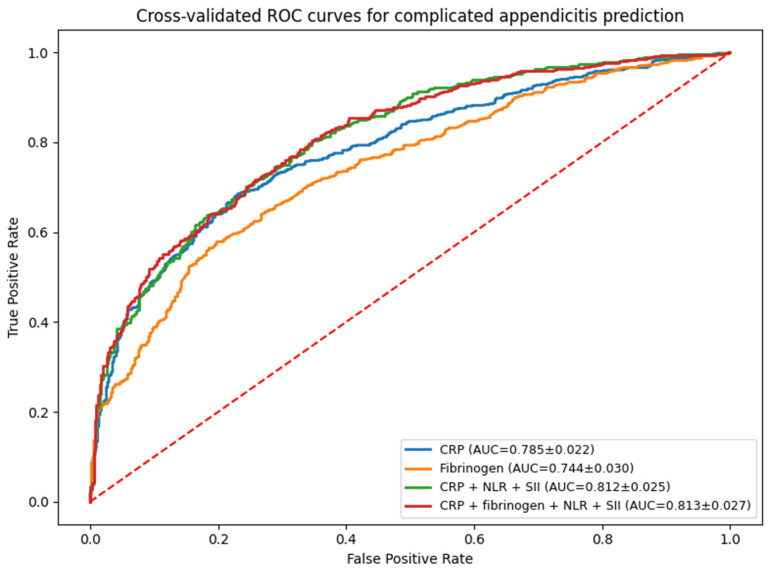
Comparative ROC curves of evaluated multivariable models.

Receiver operating characteristic (ROC) analysis showed CRP with the highest univariate AUC (0.785), followed by fibrinogen (0.744). Multivariable modelling demonstrated improved diagnostic accuracy with combined markers. The four-marker model (CRP + fibrinogen + NLR + SII) achieved the highest AUC (0.813 ± 0.027), while the simplified three-marker model (CRP + NLR + SII) was nearly equivalent (0.812 ± 0.025), suggesting fibrinogen offers only marginal incremental value beyond CRP-containing combinations. For graphical representation, see Figure 2.

Two primary multivariable logistic regression models identified independent predictors (Table 5). In the CRP-based model, log-transformed CRP (OR 2.1, 95% CI 1.9–2.4) and log-transformed SII (OR 2.3, 95% CI 1.6–3.3) were significant (*p* < 0.001); in the fibrinogen-based model, log-fibrinogen was a strong predictor (OR 21.7, 95% CI 12.3–38.1; *p* < 0.001). NLR, age, and sex were non-significant in both. Perforation-specific analysis showed fibrinogen marginally improved overall model fit (*p* = 0.044; ΔAIC = −2.06) but added no benefit for perforation prediction (*p* = 0.160). Cross-validation confirmed minimal incremental gain (ΔCV-AUC < 0.01), supporting the parsimonious three-marker model as near-optimal in this paediatric cohort.

The perforation-specific analysis revealed a clear “law of diminishing returns” with respect to fibrinogen. Although its addition marginally improved model fit when predicting complicated appendicitis overall (*p* = 0.044; ΔAIC = −2.06), it conferred no statistically significant benefit for the clinically critical outcome of perforation (*p* = 0.160). Cross-validation demonstrated minimal incremental discriminative gain across models (ΔCV-AUC < 0.01), indicating that the parsimonious three-marker combination (CRP + NLR + SII) already approaches the upper limit of achievable performance in this paediatric cohort.

To top everything off, an additional multivariable model incorporating INR alongside the primary three-marker combination (CRP + NLR + SII) was evaluated to assess its contribution to risk prediction. Inclusion of INR significantly improved model fit (likelihood ratio test χ^2^ = 9.12, *p* = 0.0025; ΔAIC = −7.12). In the adjusted analysis, INR emerged as a strong independent predictor of complicated appendicitis (OR 5.84, 95% CI 1.82–18.71; *p* = 0.003). Despite this statistical improvement, the incremental discriminative value remained negligible, with cross-validated AUC increasing by only 0.002 (0.815 ± 0.022 vs. 0.812 ± 0.025 for the base model without INR).

### 3.4. Thresholds for Clinical Applicability

To enhance practical utility, probability thresholds were derived from the multivariable logistic regression model (incorporating CRP, NLR, SII, age, and sex; *n* = 1132) and from the standalone performance of CRP.

Using the Youden index to maximize the sum of sensitivity and specificity, the optimal probability threshold for the multivariable model was 0.448. At this cut-off, the model achieved balanced diagnostic performance with a sensitivity of 0.719 and specificity of 0.756, positive predictive value (PPV) of 0.781, and negative predictive value (NPV) of 0.690 (overall accuracy = 0.736).

For scenarios prioritizing safety, such as ruling out complicated appendicitis to support non-operative management or rapid discharge in low-risk presentations, a lower probability threshold of 0.303 was selected to achieve at least 90% sensitivity. Application of this rule-out cut-off results in a sensitivity of 0.900, specificity of 0.494, PPV of 0.683, and NPV of 0.803. Cases falling below 0.303 therefore carry a low likelihood of complicated disease based on admission laboratory data alone.

Besides, a standalone high-specificity threshold for CRP was established at 92.24 mg/L to serve as a rule-in criterion. Exceeding this value delivers a specificity of 0.943 and a positive predictive value of 0.900, offering strong biochemical confirmation of complicated appendicitis despite a more modest sensitivity of 0.421. This cut-off leverages the wide availability of CRP testing and can support accelerated surgical referral when markedly elevated. All these findings can be found summarised in Table 6.

### 3.5. Clinical Application and Practical Calculation of the Risk Score

The primary multivariable model predicts the probability of complicated appendicitis using five admission variables: C-reactive protein (CRP in mg/L), Neutrophil-to-Lymphocyte Ratio (NLR), Systemic Immune-Inflammation Index (SII), age in years, and binary sex (0 = female, 1 = male). The model was trained with z-score standardization of all continuous predictors and class-weighted logistic regression. Detailed results and logic are presented before, but in summary, to minimize false negatives, a probability cut-off of 0.303 was identified, achieving ≥90% sensitivity, and at this threshold, we obtain: sensitivity 0.900, specificity 0.494, PPV: 0.683, NPV: 0.803.

Stepwise instructions for practical calculation are available in Appendix A.

### 3.6. Perforation Status Stratification

Perforation status was available for all included patients. Out of all patients, 257 (22.7%) were classified as perforated appendicitis, while 875 (77.3%) were non-perforated. When stratified by perforation status, several inflammatory biomarkers differed significantly between groups, as visible in Table 7.

## 4. Discussion

The preoperative differentiation between uncomplicated and complicated appendicitis remains a challenge in paediatric surgery.

This retrospective study of 1132 paediatric patients with confirmed acute appendicitis demonstrates significant differences in demographic, clinical, and laboratory profiles between uncomplicated and complicated cases. The observed separation across leukocyte subpopulations and acute-phase reactants aligns with the expected pathophysiology of progressive appendiceal inflammation, where neutrophil predominance and relative lymphopenia reflect innate immune activation and stress responses accompanying advanced disease.

Complicated appendicitis rates can come as high (54.8%) and possible reasons for such findings are as follows: this was a surgically managed cohort, which is likely to enrich for children with more advanced disease at presentation; our study used the broader EAES-based complicated/uncomplicated framework [5]; our complete-case design may have preferentially retained patients with more extensive admission work-up, who were also more likely to have severe clinical presentations; as a regional referral centre, our institution may receive a disproportionate number of complex cases. Across the literature, rates vary from 16.8% in developed settings, to 45–47.7% depending on pandemic context, to 75% in developing settings [36,37,38]. Perforation-only series also tend to report lower (~30%) frequencies than broader “complicated appendicitis” definitions [39].

The supplementary comparison between included and excluded patients demonstrated similar distributions of age, sex, and length of hospital stay, suggesting that the cohorts were broadly comparable in baseline characteristics. However, a difference in disease severity was observed, likely reflecting routine clinical practice in which more extensive laboratory evaluation is performed in patients with more severe or uncertain presentations. Consequently, the analytical cohort may be modestly enriched for patients with more advanced inflammatory disease, which should be considered when interpreting the biomarker performance estimates.

Among individual biomarkers, CRP demonstrated the strongest univariable discrimination for complicated appendicitis in our dataset. This finding agrees with a broad body of work describing CRP as a useful marker of advanced inflammation and perforation risk in children, particularly when interpreted alongside clinical context and other investigations [17,40].

Fibrinogen also differed significantly between groups and showed moderate univariable discrimination [41,42,43]. Unlike prior reports, which largely assessed fibrinogen in isolation, the present study formally quantifies its incremental predictive value beyond CRP and composite indices using nested models and cross-validation. However, when incorporated into multivariable models already containing CRP and SII, its incremental contribution to predictive performance was negligible, with minimal changes in AUC and no meaningful improvement in cross-validated discrimination. This pattern is biologically plausible because fibrinogen and CRP are both acute-phase reactants whose hepatic synthesis is driven predominantly by interleukin-6 (IL-6) through shared JAK-STAT3 signalling, increasing the likelihood of correlated rather than complementary diagnostic signals [44,45]. When two markers share a common upstream driver of this magnitude, the variance in outcome that fibrinogen could explain is already captured—and captured more precisely, given CRP’s stronger univariable AUC (0.785 vs. 0.744)—by CRP alone, a form of collinearity that reduces incremental predictive value irrespective of statistical significance in model-fit tests.

Composite indices further enhanced differentiation, with higher NLR (10.38 vs. 5.38) and SII (3094.92 vs. 1560.39; *p* < 0.001) in complicated cases. Meta-analyses support NLR’s moderate diagnostic value for paediatric appendicitis (AUC 0.86, sensitivity 0.82, specificity 0.76), while emerging data on SII indicate strong performance (AUC 0.95 for overall appendicitis, moderate for complicated) [46,47,48]. Our multivariable integration aligns with studies combining CRP with NLR/SII, improving AUC to 0.80–0.90. Novel ratios like CLR (CRP/lymphocytes) have shown comparable AUC (0.772) for complicated disease, suggesting potential avenues for refinement. The negligible incremental discriminative gain (ΔCV-AUC ≤ 0.002) and lack of added benefit for perforation-specific prediction underscore fibrinogen’s and INR’s limited independent utility in this appendicitis stratification context. The key novelty of the present analysis lies not in the individual evaluation of these markers in isolation, but in the rigorous quantification of their incremental contributions within a unified multivariable framework: the three-marker combination of CRP, NLR, and SII already approaches the practical performance ceiling achievable from admission laboratory data, and neither fibrinogen nor INR meaningfully exceeds this ceiling in cross-validated testing.

An additional exploratory stratification according to perforation status demonstrated broadly similar patterns of inflammatory marker distribution. Patients with perforated appendicitis showed higher levels of CRP, fibrinogen, NLR, PLR, and SII, alongside lower LMR values compared with non-perforated cases, reflecting a greater systemic inflammatory burden accompanying advanced disease. However, because complicated appendicitis in our cohort also included severe non-perforated forms such as gangrenous inflammation, peri-appendiceal abscess, or inflammatory mass formation, perforation alone does not fully capture the spectrum of advanced disease.

A useful comparator is the recent single-centre retrospective study by Puputti et al., which analysed 1149 surgically treated paediatric appendicitis cases and focused on CRP combined with imaging to identify uncomplicated outcomes [40]. In that cohort, complicated cases had substantially higher preoperative CRP (reported as 133 ± 83 mg/L vs. 47.2 ± 46.3 mg/L in uncomplicated outcomes), and among children with uncomplicated imaging findings, a CRP cut-off ≤58 mg/L yielded an AUROC of 0.819 (95% CI 0.78–0.86) with 85.7% sensitivity and 90.0% specificity for predicting uncomplicated outcome; only 20/450 (4.4%) with uncomplicated imaging and CRP ≤58 mg/L nevertheless had a complicated outcome. In comparison, our work similarly confirms the strong discriminatory value of CRP but does not rely on imaging-dependent stratification by incorporating CBC-derived composite indices (notably SII) into multivariable modelling, enabling probability-based thresholds that can be applied using admission laboratory data when imaging findings are equivocal, unavailable, or variably reported.

Although abdominal ultrasound remains indispensable in the diagnostic work-up of suspected paediatric appendicitis and is recommended as first-line imaging in children, cross-sectional imaging is often pursued when ultrasound is equivocal or non-diagnostic [49,50,51]. In this context, the probability-based laboratory model developed in our study may help reduce reliance on computed tomography (CT)—particularly in borderline presentations—by providing an objective, admission-laboratory estimate of complicated disease risk that can support triage and escalation decisions while awaiting repeat ultrasound, senior review, or MRI where available. This is clinically relevant because CT exposes children to ionizing radiation, and multiple high-quality sources highlight that radiation-related cancer risk is higher in children than adults for the same CT exposure; consequently, strategies that safely limit CT use are strongly aligned with paediatric imaging safety principles [52].

Laboratory-based probability estimates should be considered as supportive information integrated with clinical assessment and established decision tools rather than as independent decision-making instruments. The probability thresholds explored in this study may therefore complement existing diagnostic elements, including ultrasound findings and commonly used clinical scores such as the Paediatric Appendicitis Score (PAS) or the Appendicitis Inflammatory Response (AIR) score [53,54]. In practical terms, a low predicted probability could support short-term observation, repeat ultrasound, or outpatient reassessment in clinically stable patients, whereas a high predicted probability or markedly elevated CRP may strengthen the case for expedited surgical review or advanced imaging. Intermediate-risk results would still require integration with clinical judgement and imaging findings, reinforcing the role of the model as an adjunctive triage tool rather than a replacement for established diagnostic pathways.

A key contribution of this study is the evaluation of cell-derived composite indices derived from routine CBC parameters. Indices such as NLR, PLR, LMR, and SII have attracted growing attention as they summarize the balance of innate (neutrophil/platelet) and adaptive (lymphocyte) components of inflammation, potentially improving signal-to-noise compared with single counts. Our findings support this concept, with SII showing consistent association with complicated appendicitis in multivariable analyses, while some indices offered weaker or inconsistent incremental value once CRP and SII were included. These results align with the broader movement toward biomarker combinations and algorithmic stratification in paediatric appendicitis, including machine learning–based approaches that integrate laboratory features to improve discrimination, though published model performances vary widely depending on outcome definitions, cohort composition, and validation strategy.

The standalone CRP rule-in threshold (≥92.24 mg/L; specificity 0.943, PPV 0.900) remains a simple, single-biomarker alternative when rapid multivariable calculation is not feasible, but it should not replace the full model when all variables are available. Together, these findings define two clinically applicable probability thresholds for the multivariable model: a lower threshold favouring sensitivity (0.303) and a balanced diagnostic threshold (0.448), alongside a high-specificity CRP rule-in value for biochemical confirmation. A complete-case analytical strategy was used to ensure consistent laboratory data across predictors, minimizing bias introduced by disparate laboratory reporting with the trade-off of inclusion bias [33].

These cut-offs were derived from the development dataset and should be interpreted as exploratory thresholds illustrating potential clinical operating points for risk stratification, “rule-out,” and “rule-in” strategies. Such thresholds illustrate a balanced operating point within the current dataset and may serve as a hypothesis-generating reference for future validation studies. Specifically, patients with predicted probability < 0.303 showed a low likelihood of complicated appendicitis within this dataset, suggesting a potential rule-out operating point that requires validation in independent cohorts before clinical adoption.

Clinically, our results advocate a tiered approach: initial CRP for quick rule-in in high-suspicion cases, followed by multivariable probability for nuanced stratification. This could integrate with scores like PAS or AIR, which show moderate AUC (0.60–0.70) but benefit from biomarker augmentation. Implementation may lower costs and radiation exposure by reducing unnecessary imaging, especially in resource-limited settings.

Several limitations should be acknowledged. First, the retrospective design is inherently vulnerable to unmeasured confounding, and the single-centre setting may limit generalizability across regions with different referral patterns, imaging availability, and laboratory workflows. Second, a complete-case approach was used for key laboratory variables, which improves internal consistency for modelling but can introduce bias if missingness is not completely at random; the high contribution of fibrinogen missingness may reflect operational ordering practices rather than patient biology. It should also be noted that fibrinogen is not universally included in the admission work-up for suspected appendicitis; at our institution, it forms part of a standard coagulation panel obtained routinely in all children presenting with acute abdominal pathology, though this practice may differ across centres. In settings where fibrinogen is ordered selectively, its practical applicability as a predictive marker would be further limited, reinforcing the clinical utility of the parsimonious CRP + NLR + SII model, which relies exclusively on markers universally available from routine admission testing. Third, severity classification relied on retrospective interpretation of operative, clinical, and histopathological documentation mapped to the EAES consensus framework [5]. Although this reflects routine clinical practice, variability in descriptive terminology may arise, particularly for borderline entities such as phlegmonous disease and localised contamination. While concordance between intraoperative and histopathological findings is generally high for unambiguous presentations such as frank perforation or abscess, moderate disagreement may occur for borderline entities; in our cohort, such discordant cases were resolved by defaulting to the more severe classification, which minimises underclassification risk but may introduce a small degree of upward severity bias.

Fourth, probability cut-offs were derived from the same development dataset used to fit the multivariable model; although discrimination was internally assessed using cross-validation, external validation was not performed; therefore, calibration and clinical transportability of the proposed thresholds require confirmation in independent cohorts. Finally, the model uses routinely available laboratory predictors and basic demographics only; the absence of imaging findings, symptom duration, and clinical examination features likely caps achievable discrimination and may explain the modest incremental gains observed when adding additional laboratory variables.

Future work should prioritize (i) prospective or multi-centre validation of the multivariable model and its thresholds, including calibration assessment; (ii) evaluation of combined clinical–laboratory–imaging models consistent with current diagnostic pathways; and (iii) decision-analytic assessment of how a high-sensitivity “rule-out” threshold or high-specificity “rule-in” threshold changes management decisions, resource utilization, and outcomes. In addition, exploring structured documentation or standardized operative/histopathologic severity grading could reduce label variability and improve model robustness across institutions.

## 5. Conclusions

In this retrospective single-centre cohort of paediatric patients with confirmed acute appendicitis, routinely obtained admission laboratory parameters showed clear separation between complicated and uncomplicated disease. Complicated appendicitis was consistently associated with higher inflammatory burden across conventional biomarkers and with characteristic shifts in leukocyte subpopulations, which were captured by CBC-derived composite indices.

Fibrinogen provided little additional diagnostic value for predicting complicated appendicitis once CRP and SII were included in multivariable models. Although fibrinogen was independently associated with complicated disease, its incremental contribution to overall model discrimination was negligible, suggesting that routine fibrinogen testing is unlikely to meaningfully improve clinical risk stratification when CRP-based markers are already available, though prospective multi-centre validation is required before this finding can inform test-ordering practice. In contrast, CRP demonstrated the strongest univariable discriminatory performance and, together with SII, remained independently associated with complicated appendicitis in multivariable analyses.

The multivariable probability model incorporating CRP, NLR, SII, age, and sex achieved good discriminative performance and enabled the derivation of clinically oriented thresholds, including a high-sensitivity cut-off suited to rule-out–focused triage and a balanced threshold for general risk stratification. Together, these findings illustrate two candidate probability thresholds for the multivariable model—a high-sensitivity rule-out operating point and a balanced diagnostic threshold—which should be viewed as hypothesis-generating until externally validated.

## Figures and Tables

**Table 1:** Characteristics of the study population.

Variable	Whole Cohort (*n* = 1132)	Complicated (*n* = 620)	Uncomplicated (*n* = 512)	*p*-Value
Age (years)				0.0101
Mean (SD)	11.23	10.93	11.58	
Median (IQR)	11.12 (8.6–14.4)	10.67 (8.1–14.4)	11.50 (9.1–14.4)	
Age (months)				
Median (IQR)	133.5 (103.0–173.0)	128.0 (96.8–173.0)	138.0 (109.0–173.2)	
Sex, *n* (%)				0.0514
Male	719 (63.5%)	410 (66.1%)	309 (60.4%)	
Female	413 (36.5%)	210 (33.9%)	203 (39.6%)	
Length of Stay (days)				<0.0001
Mean (SD)	6.73	8.29	4.84	
Median (IQR)	6.0 (4.0–8.0)	8.0 (6.0–9.0)	4.0 (3.0–6.0)	
Appendicectomy	1040 (91.9%)	585 (94.4%)	455 (88.9%)	0.0011

**Table 2:** Comparative Analysis of Haematological Parameters.

Parameter	Whole Cohort Median (IQR)	Complicated Median (IQR)	Uncomplicated Median (IQR)	Whole Mean ± SD	*p*-Value
WBC	16.08 (12.1–20.1)	17.71 (13.9–21.4)	14.05 (10.7–17.5)	16.33 ± 5.98	<0.001
HGB	12.90 (12.2–13.7)	12.90 (12.0–13.7)	13.00 (12.3–13.8)	12.94 ± 1.39	0.041
Neutrophils	12.84 (9.1–16.9)	14.50 (11.1–18.2)	10.89 (7.4–14.8)	13.18 ± 5.78	<0.001
Lymphocytes	1.59 (1.1–2.3)	1.39 (1.0–2.0)	1.92 (1.2–2.6)	1.78 ± 0.92	<0.001
Monocytes	1.14 (0.8–1.6)	1.31 (0.9–1.8)	0.98 (0.7–1.3)	1.26 ± 0.71	<0.001
Platelets	297.00 (249.8–345.0)	304.00 (250.0–355.2)	292.00 (249.0–335.0)	304.37 ± 84.21	0.031
INR	1.16 (1.1–1.3)	1.21 (1.1–1.3)	1.11 (1.1–1.2)	1.19 ± 0.16	<0.001

All haematological parameters are reported in conventional units: WBC, neutrophils, lymphocytes, and monocytes in ×10^3^/μL; HGB in g/dL; platelets in ×10^3^/μL; INR as unitless ratio.

**Table 3:** Distribution of Inflammatory Biomarkers.

Parameter	Complicated Median (IQR)	Uncomplicated Median (IQR)	*p*-Value
CRP	75.39 (28.9–144.4)	15.04 (4.0–36.2)	<0.001
Fibrinogen	433.00 (362.8–513.0)	345.00 (287.5–402.0)	<0.001

CRP, C-reactive protein.

**Table 4:** Composite Inflammatory Indices.

Parameter	Complicated Median (IQR)	Uncomplicated Median (IQR)	*p*-Value
NLR	10.38 (6.3–15.9)	5.38 (3.1–10.0)	<0.001
PLR	213.54 (151.9–301.5)	152.83 (111.5–223.7)	<0.001
LMR	1.11 (0.8–1.6)	1.85 (1.2–2.9)	<0.001
SII	3094.92 (1818.0–4801.2)	1560.39 (882.0–3038.5)	<0.001

NLR, neutrophil-to-lymphocyte ratio; PLR, platelet-to-lymphocyte ratio; LMR, lymphocyte-to-monocyte ratio; SII, systemic immune-inflammation index.

**Table 5:** Multivariable Predictors of Complicated Appendicitis.

Variable	CRP-Based Model OR (95% CI)	*p*-Value	Fibrinogen-Based Model OR (95% CI)	*p*-Value
log(Biomarker) *	2.1 (1.9–2.4)	<0.001	21.7 (12.3–38.1)	<0.001
NLR	1.0 (1.0–1.0)	0.731	1.0 (1.0–1.0)	0.900
log(SII)	2.3 (1.6–3.3)	<0.001	2.2 (1.5–3.0)	<0.001
Age	1.0 (1.0–1.0)	0.937	1.0 (1.0–1.0)	0.685
Sex (Male)	1.2 (0.9–1.6)	0.293	1.2 (0.9–1.6)	0.166
Fit Statistics	AIC: 1209.70; R^2^: 0.23	—	AIC: 1291.54; R^2^: 0.18	—

* Biomarker refers to CRP in the first model and fibrinogen in the second. All models were adjusted for age and sex. NLR, neutrophil-to-lymphocyte ratio. SII, systemic immune-inflammation index.

**Table 6:** Summary of Practical Diagnostic Thresholds and Clinical Performance.

Strategy	Threshold	Clinical Profile	Sensitivity	Specificity	PPV	NPV
Balanced Model	0.448 (probability)	Balanced diagnostic performance	0.719	0.756	0.781	0.690
High-Sensitivity	0.303 (probability)	Optimal for rule-out strategy	0.900	0.494	0.683	0.803
CRP Rule-In	≥92.24 mg/L	High specificity, rule-in oriented	0.421	0.943	0.900	0.574

**Table 7:** Distribution of inflammatory biomarkers according to perforation status.

Biomarker	Perforated (*n* = 257) Median (IQR)	Non-Perforated (*n* = 875) Median (IQR)	*p*-Value
CRP (mg/L)	113.46 (57.10–185.27)	26.13 (7.30–65.10)	<0.001
Fibrinogen (mg/dL)	464.00 (407.00–550.00)	366.00 (304.00–433.00)	<0.001
NLR	11.46 (6.98–16.73)	7.13 (3.87–12.42)	<0.001
PLR	239.20 (173.68–322.03)	170.00 (122.18–248.42)	<0.001
LMR	1.01 (0.71–1.44)	1.51 (1.00–2.33)	<0.001
SII	3338.59 (1877.33–5078.82)	2125.21 (1089.66–3791.75)	<0.001

## Data Availability

De-identified clinical and laboratory data supporting the findings of this study are available from the corresponding authors upon reasonable request, subject to institutional data-sharing policies.

## Abbreviations

The following abbreviations are used in this manuscript:

AA	Acute appendicitis
AASI	Acute Appendicitis Severity Index
AIC	Akaike Information Criterion
AIR	Appendicitis Inflammatory Response
AUC	Area under the curve
CA	Complicated appendicitis
CI	Confidence interval
CRP	C-reactive protein
CT	Computed tomography
EAES	European Association for Endoscopic Surgery
EHR	Electronic health records
GDPR	General Data Protection Regulation
HGB	Hemoglobin
ICD-10	International Classification of Diseases, 10th Revision
INR	International Normalized Ratio
PPV	Positive predictive value
ROC	Receiver Operating Characteristic
SD	Standard deviation
SII	Systemic immune-inflammation index
UA	Uncomplicated appendicitis
US	Ultrasound
WBC	White blood cell count
WSES	World Society of Emergency Surgery

## Appendix A

In clinical practice, the predicted probability (p) of complicated appendicitis can be calculated manually as follows:

Compute the standardized (z-score) values using the training-set means and standard deviations (these values should be reported to enable external use of the model):

z_CRP = (CRP—mean_CRP)/sd_CRP
z_NLR = (NLR—mean_NLR)/sd_NLR
z_SII = (SII—mean_SII)/sd_SII
z_age = (age—mean_age)/sd_age
z_sex = (sex_binary—mean_sex)/sd_sex (note: this adjustment is usually small because sex is binary)

Calculate the linear predictor (log-odds):

logit(p) = β_0_ + β_1_ × z_CRP + β_2_ × z_NLR + β_3_ × z_SII + β_4_ × z_age + β_5_ × z_sex
(where β_0_ is the intercept and β_1_–β_5_ are the model coefficients, which should be reported)

Convert the log-odds to probability:

p = 1/(1 + exp(–logit(p)))

The resulting probability p (ranging from 0 to 1) is then compared to the chosen clinical threshold:

p < 0.303 → high-sensitivity rule-out (sensitivity 0.900, NPV 0.803); supports consideration of non-operative management or expedited discharge in low-risk, equivocal cases (pending full clinical assessment).
p ≥ 0.448 → balanced threshold (sensitivity 0.719, specificity 0.756, PPV 0.781); suitable for general risk stratification and decision support.
